# Epidemiological and clinical characteristics of respiratory syncytial virus and influenza infections in hospitalized children before and during the COVID‐19 pandemic in Central China

**DOI:** 10.1111/irv.13103

**Published:** 2023-02-02

**Authors:** Lingshuang Ren, Li Lin, Hua Zhang, Qianli Wang, Yibing Cheng, Qin Liu, Bing Fang, Linsen Xie, Meng Wang, Juan Yang, Jinxin Guo, Tianchen Zhang, Hongkai Lian, Jiangtao Wang, Hongjie Yu

**Affiliations:** ^1^ School of Public Health Fudan University, Key Laboratory of Public Health Safety, Ministry of Education Shanghai China; ^2^ Children's Hospital Affiliated to Zhengzhou University Henan Children's Hospital Zhengzhou China; ^3^ Zhengzhou Central Hospital Affiliated to Zhengzhou University Zhengzhou China; ^4^ Shanghai Institute of Infectious Disease and Biosecurity Fudan University Shanghai China; ^5^ Division of Infectious Disease Jiangxi Province Center for Disease Control and Prevention Nanchang China

**Keywords:** child, COVID‐19, hospitalization, influenza, respiratory syncytial virus

## Abstract

**Background:**

Globally, the epidemiology of non‐SARS‐CoV‐2 respiratory viruses like respiratory syncytial virus (RSV) and influenza virus was remarkably influenced by the implementation of non‐pharmacological interventions (NPIs) during the COVID‐19 pandemic. Our study explored the epidemiological and clinical characteristics of pediatric patients hospitalized with RSV or influenza infection before and during the pandemic after relaxation of NPIs in central China.

**Methods:**

This hospital‐based prospective case‐series study screened pediatric inpatients (age ≤ 14 years) enrolled with acute respiratory infections (ARI) for RSV or influenza infection from 2018 to 2021. The changes in positivity rates of viral detection, epidemiological, and clinical characteristics were analyzed and compared.

**Results:**

Median ages of all eligible ARI patients from 2018–2019 were younger than those from 2020–2021, so were ages of cases infected with RSV or influenza (RSV: 4.2 months vs. 7.2 months; influenza: 27.3 months vs. 37.0 months). Where the positivity rate for influenza was considerably decreased in 2020–2021 (1.4%, 27/1964) as compared with 2018–2019 (2.9%, 94/3275, P < 0.05), it was increased for RSV (11.4% [372/3275] vs. 13.3% [262/1964], P < 0.05) in the same period. The number of severe cases for both RSV and influenza infection were also decreased in 2020–2021 compared with 2018–2019.

**Conclusions:**

The implemented NPIs have had varied impacts on common respiratory viruses. A more effective prevention strategy for RSV infections in childhood is needed.

## 1 INTRODUCTION

Since December 2019, the world has been experiencing coronavirus disease 2019 (COVID‐19) pandemic. In China, the nationwide implemented non‐pharmaceutical interventions (NPIs) were relaxed in April 2020 when the transmission of COVID‐19 was under control.^1^ These huge public health interventions which caused broad‐spectrum changes in human behavior also affected the activity of other seasonal respiratory viruses such as respiratory syncytial virus (RSV) and influenza virus, as they share similar routes of transmission as severe acute respiratory syndrome‐coronavirus‐2 (SARS‐CoV‐2). Acute respiratory infections (ARI) especially viral lower respiratory tract infections, such as pneumonia and bronchiolitis caused by influenza and RSV are the leading causes of morbidity and mortality in children younger than 5 years.^2^

After implementation of NPIs, a remarkable reduction in the circulation of influenza was commonly observed in almost all temperate zones and some tropical areas of the world.^3^, ^4^ However, this changed activity pattern of these viruses was not synchronous and varied from place to place. For example, in the winter of 2020, a low transmission of RSV and a delayed inter‐seasonal resurgence was observed in Australia, Europe, and America.^5^, ^6^, ^7^, ^8^, ^9^ There were several hospital‐based studies from eastern and southern provinces of mainland China, which noted a high activity of RSV with a typical epidemic season.^10^, ^11^, ^12^ However, no such studies are available, which covered the epidemiological and clinical information of patients from central China.

In this study, we aimed to report the impact of COVID‐19 NPIs on the epidemiology and clinical characteristics of RSV and influenza infections in hospitalized pediatric patients from central China. Based on this prospective case‐series study, multiple comparisons were made between these infections before and during the pandemic.

## 2 METHODS

### 2.1 Study design and participants

This hospital‐based prospective case‐series study took place in Henan Children's Hospital, Zhengzhou. Zhengzhou is the capital city of Henan Province in central China with a permanent resident population of 2.4 million children aged 0–14 years.^13^ The first study period lasted from December 1, 2018, to November 30, 2019 (study year 2018–2019); and the second study period began on December 29, 2020 till October 20, 2021 (study year 2020–2021). The study had to be suspended for a year from December 2019 to December 2020 due to COVID‐19 pandemic.

The study was conducted on the largest campus (East Campus) of Henan Children's Hospital where three‐fourths of all the inpatients were admitted. Participants were enrolled in three wards: general emergency ward, infant ward, and pediatric intensive care unit (PICU) as per the severity of illness. The PICU and general emergency ward admitted children aged 6 months to 14 years old, and infants (28 days to 6 months) were admitted to the infant ward.

All inpatients aged 14 years or less were screened for ARI. The inclusion criteria for an ARI patient needed: (i) admission to hospital with new illness onset of suspected ARI within 7 days for patients aged >3 months and an onset within 10 days for infants aged ≤3 months; (ii) manifestation of at least one of respiratory symptoms including cough, sore throat, rhinorrhea, and congestion of the nasal mucosa or pharynx; (iii) development of acute fever with an axillary temperature measured ≥37.5°C for patients aged >3 months. The date of illness onset was defined as the day when the symptom was first noticed.

### 2.2 Definitions

Acute respiratory infection (ARI): A comprising infection caused by infectious pathogens such as bacteria and viruses leading to acute inflammation in any part of respiratory system. It is divided into upper or lower respiratory infection based on anatomy.^14^

Severe RSV or influenza illness: Illness requiring respiratory‐support with non‐invasive positive pressure ventilation (such as high flow nasal cannula, continuous positive airway pressure [CPAP], bi‐level positive airway pressure [BIPAP]), or invasive ventilation (endotracheal intubation or ECMO), ICU admission or event of in‐hospital death.^15^

Relaxation of NPIs: A post‐epidemic state in which all provinces eased their public health measures with relaxation of human mobility, reopening of schools and businesses, resumption of recreational activities while maintaining social distancing.^1^

Pre‐term birth: Babies are born alive before 37 weeks of pregnancy are completed.

### 2.3 Sample collection and laboratory test

The enrolled ARI patients were approached for collection of respiratory specimens (throat swabs) within 24 h of hospitalization and then tested within 48 h for RSV and influenza infection by real time‐polymerase chain reaction (RT‐PCR). Methods for influenza and RSV detection are detailed in Supporting Information.

### 2.4 Data collection

Demographics, medical history, underlying conditions such as pre‐term birth, and clinical information (including clinical manifestations, laboratory findings, chest radiography, and treatment) were extracted from electronic medical records of the hospital. The extracted data were recorded using a standardized data dictionary and REDCap system (Research Electronic Data Capture, Vanderbilt University, Nashville, Tennessee, USA) or EpiData v3.1. The data were checked for completeness and accuracy by the investigators. Information on birth weight and feeding patterns in the first year of life was also recorded if available.

### 2.5 Statistical analysis

Continuous variables were described with medians and interquartile ranges (IQR) and categorical variables were described with frequencies and percentages. Comparisons of RSV or influenza infection characteristics were made using chi‐square test. In case of limited data availability, Fisher's exact test was used, whereas non‐normal distributed continuous variables were compared using Mann–Whitney–Wilcoxon test. Multivariable logistic regression models were developed to identify the potential factors associated with severe illness, calculation of odds ratios (ORs), and 95% confidence intervals. We first used a univariate regression model for candidate variable selection (P < 0.1), then performed a forward stepwise model selection based on Akaike's information criterion (AIC) and likelihood ratio test to select the explanatory variables in final model. Candidate variables are listed in the Supporting Information. A P value of <0.05 was considered statistically significant. All statistical analyses were performed in R v3.6.3 (R Foundation for Statistical Computing, Vienna, Austria).

## 3 RESULTS

### 3.1 Characteristics of study population

**FIGURE 1:**
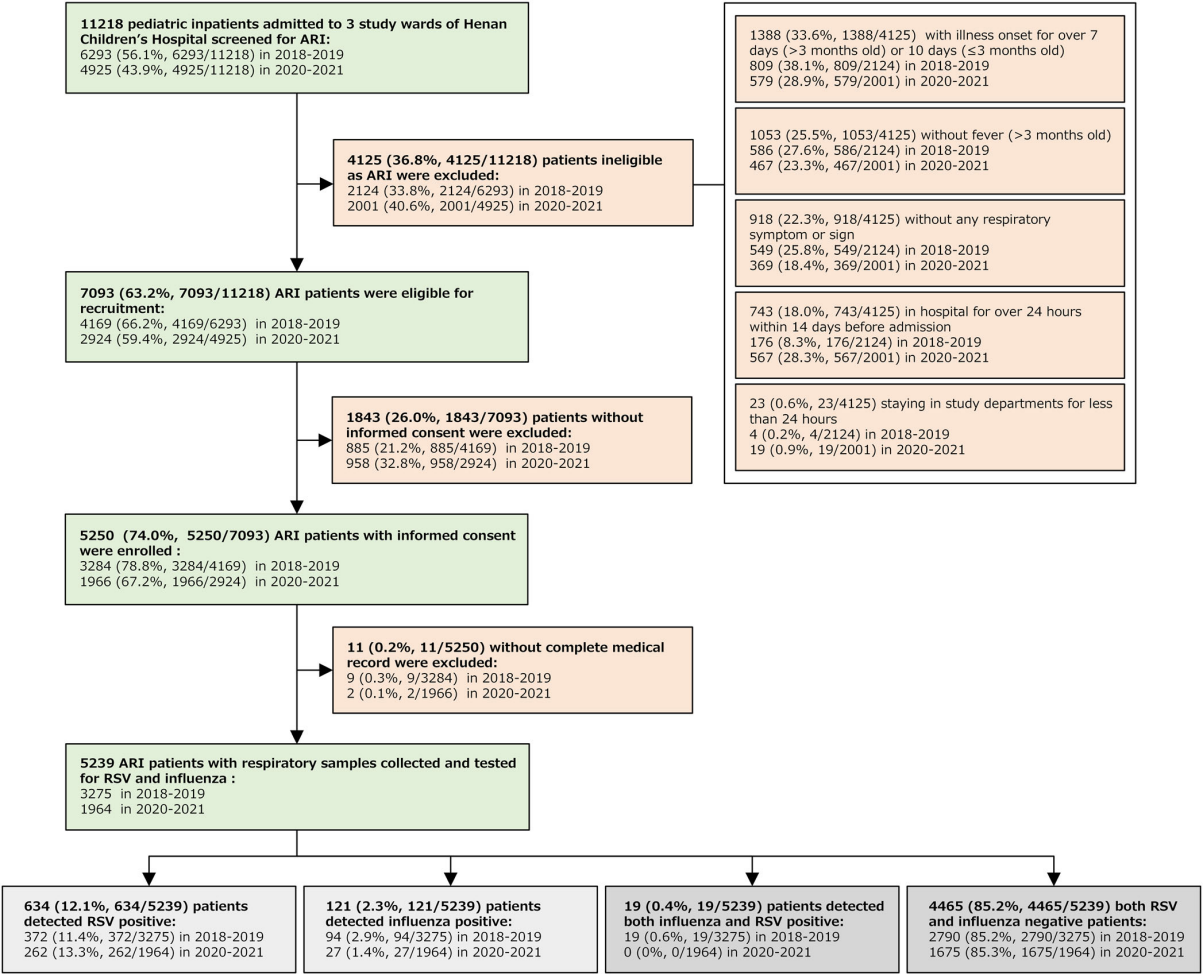
Flow chart for the enrollment of pediatric ARI inpatients for detection of RSV and influenza in 2018–2019 and 2020–2021 study. The 19 RSV and influenza co‐infected patients were removed from further analysis, so the box is marked gray, along with RSV and influenza‐negative ARI patients.

Throughout the study years, a total of 7093 eligible ARI patients were admitted in the three wards. Out of these eligible patients, 5250 (74.0%) patients with an informed consent were enrolled in this study. Remaining 1843 (26.0%) patients were excluded from the study on guardians' request. The main reason behind refusal of participation was the guardians' unwillingness for their children to receive the throat swab sampling procedure. Additional 11 patients were excluded due to incomplete medical records. In the end, 5239 patients remained in study for the screening of influenza and RSV infection. These 5239 patients included 3275 patients from 2018–2019 and 1964 patients from 2020–2021 (Figure 1 and Table S1).

Generally, the median age of ARI patients in 2018–2019 was younger than those enrolled in 2020–2021 (8.0 months, IQR: 2.6–31.1 vs. 19.0 months, IQR: 6.4–43.0; P < 0.05) (Table 1). Patients from 2020–2021 had a higher proportion of local residents (76.6%) than those from 2018–2019 (63.1%). The proportion of patients with underlying conditions also decreased from 13.8% to 7.9% (P < 0.05) in 2020–2021 (Table 1). The most common complications with ARI patients were pneumonia, bronchitis, and sepsis. These complications also showed a significant difference between both study years (pneumonia: 62.8% vs. 52.9%; bronchitis: 16.4% vs. 26.3%; sepsis: 7.6% vs. 3.0%) (not shown in tables).

**TABLE 1:** Demographic characteristics and underlying conditions of patients.

Characteristic	Enrolled ARI patients			RSV‐infected patients			Influenza‐infected patients		
	Total	2018–2019	2020–2021	Total	2018–2019	2020–2021	Total	2018–2019	2020–2021
	(n = 5239)	(n = 3275)	(n = 1964)	(n = 634)	(n = 372)	(n = 262)	(n = 121)	(n = 94)	(n = 27)
Age, month, median (IQR)	12.0 (3.0–37.2)	8.0 (2.6–31.1)	19.0 (6.4–43.0)	5.4 (2.4–15.3)	4.2 (2.3–12.6)	7.2 (2.7–18.0)	29.1 (13.0–60.0)	27.3 (13.1–52.0)	37.0 (12.0–82.5)
Age group									
0–1 month	665 (12.7)	516 (15.8)	149 (7.6)	109 (17.2)	71 (19.1)	38 (14.5)	4 (3.3)	3 (3.2)	1 (3.7)
2–3 months	952 (18.2)	714 (21.8)	238 (12.1)	169 (26.7)	112 (30.1)	57 (21.8)	9 (7.4)	7 (7.4)	2 (7.4)
4–5 months	317 (6.1)	231 (7.1)	86 (4.4)	56 (8.8)	36 (9.7)	20 (7.6)	1 (0.8)	1 (1.1)	0 (0)
6–11 months	689 (13.2)	430 (13.1)	259 (13.2)	92 (14.5)	55 (14.8)	37 (14.1)	16 (13.2)	12 (12.8)	4 (14.8)
12–23 months	791 (15.1)	433 (13.2)	358 (18.2)	117 (18.5)	55 (14.8)	62 (23.7)	23 (19.0)	21 (22.3)	2 (7.4)
24–59 months	1282 (24.5)	664 (20.3)	618 (31.5)	85 (13.4)	38 (10.2)	47 (17.9)	37 (30.6)	31 (33.0)	6 (22.2)
≥5 years	543 (10.4)	287 (8.8)	256 (13.0)	6 (0.9)	5 (1.3)	1 (0.4)	31 (25.6)	19 (20.2)	12 (44.4)
Male sex	3136 (59.9)	1982 (60.5)	1154 (58.8)	380 (59.9)	227 (61.0)	153 (58.4)	71 (58.7)	51 (54.3)	20 (74.1)
Residence									
Zhengzhou City	3571 (68.2)	2066 (63.1)	1505 (76.6)	457 (72.1)	242 (65.1)	215 (82.1)	76 (62.8)	55 (58.5)	21 (77.8)
Outside Zhengzhou City in Henan Province	1583 (30.2)	1144 (34.9)	439 (22.4)	166 (26.2)	123 (33.1)	43 (16.4)	41 (33.9)	35 (37.2)	6 (22.2)
Outside Henan Province	83 (1.6)	63 (1.9)	20 (1.0)	10 (1.6)	6 (1.6)	4 (1.5)	3 (2.5)	3 (3.2)	0 (0)
Missing data^a^	2 (0.0)	2 (0.1)	0 (0)	1 (0.2)	1 (0.3)	0 (0)	1 (0.8)	1 (1.1)	0 (0)
Underlying conditions									
Yes	606 (11.6)	451 (13.8)	155 (7.9)	68 (10.7)	48 (12.9)	20 (7.6)	15 (12.4)	13 (13.8)	2 (7.4)
No	4633 (88.4)	2824 (86.2)	1809 (92.1)	566 (89.3)	324 (87.1)	242 (92.4)	106 (87.6)	81 (86.2)	25 (92.6)

*Note*: Figures are numbers (%) unless stated otherwise. Characteristics were compared in ARI or RSV/influenza positive patients between 2018–2019 and 2020–2021.

Abbreviations: ARI, acute respiratory infections; IQR, interquartile range; RSV, respiratory syncytial virus.

^a^ Inpatients' addresses were unfilled.

### 3.2 Detection of RSV and influenza

Among 3275 patients from study year 2018–2019, 485 (14.8%) cases were found to be caused by RSV or influenza. These included 372 (11.4%) cases of RSV, 94 (2.9%) cases of influenza, and 19 (0.6%) cases of co‐infection. Since our focus was on the single viral pathogen infected patients, the data from 19 cases of co‐infection were excluded. The remaining 2790 (85.2%) cases were negative for RSV as well as influenza. In the second study year 2020–2021, there were 289 (14.7%) cases of RSV or influenza infection. These included 262 (13.3%) cases of RSV and 27 (1.4%) cases of influenza. No cases of co‐infection were detected in this study year (Figure 1). Remaining 1675 (85.3%) patients were found to be negative for both RSV and influenza. The rate of influenza infections decreased by 51.7% (from 2.9% in 2018–2019 to 1.4% in 2020–2021), while the rate remained high for RSV infections (11.4% in 2018–2019 vs. 13.3% in 2020–2021).

**FIGURE 2:**
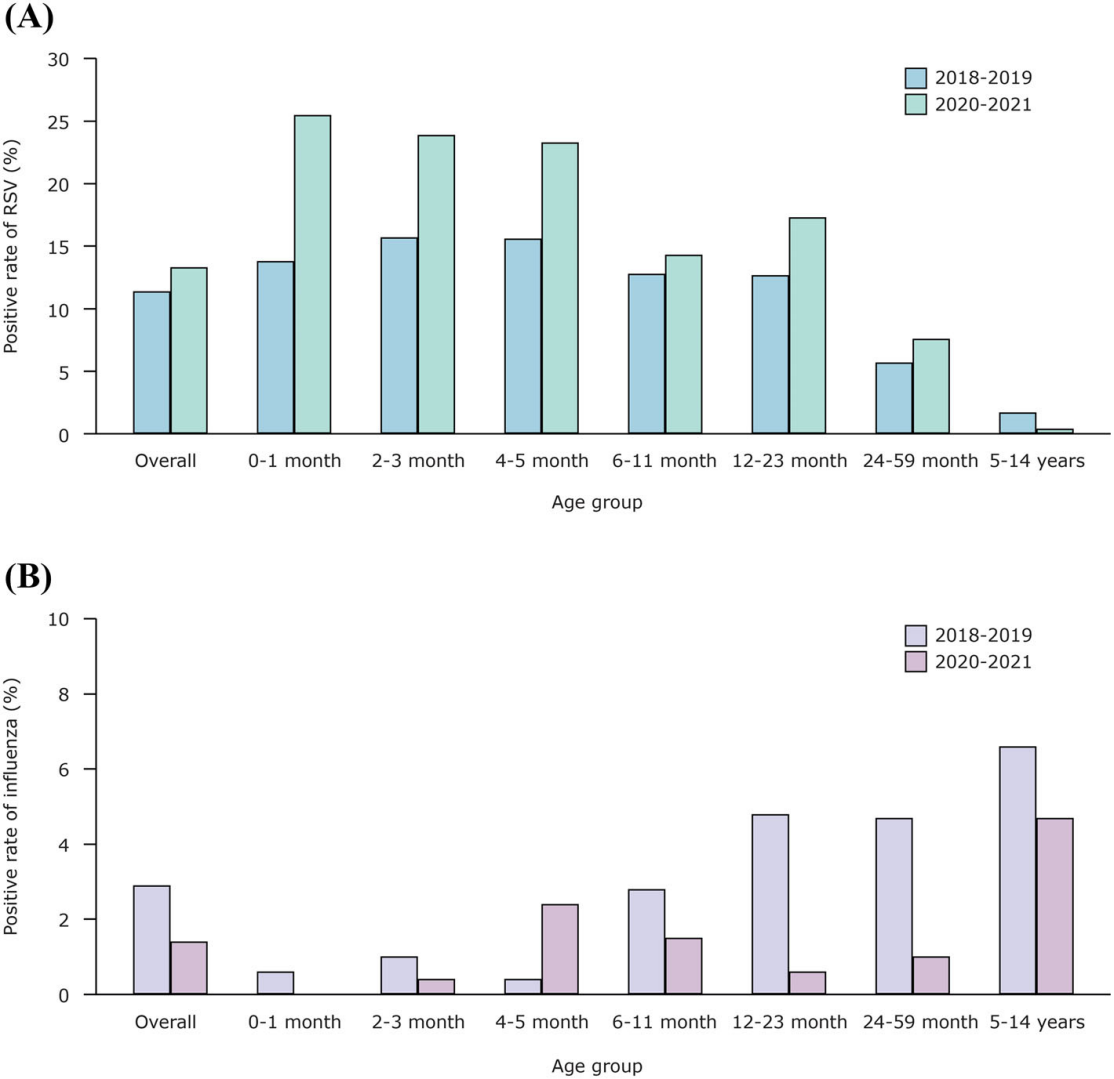
Age distribution of positive rates of RSV and influenza‐infected patients in 2018–2019 and 2020–2021 study years. The positive rates of each age subgroup were compared between study year 2018–2019 and 2020–2021. Study year 2018–2019 began on December 1, 2018 and ended on November 30, 2019; study year 2020–2021 began on December 29, 2020, and ended on October 20, 2021. (A) RSV‐infected patients; (B) influenza‐infected patients.

As shown in Figure 2, the positivity rate of influenza increased with an increase in age of children, whereas it decreased for RSV with an increase in age. In 2020–2021, the RSV positivity rates remained high in majority of age groups, especially in the group of infants <4 months (P < 0.05) (Table S2), whereas the rate was lower in the group of ≥5‐year‐olds in 2020–2021 than that of 2018–2019 without any significant differences. In case of influenza, the positivity rates remained low in 2020–2021 across all age groups except the group of 4–5 months old.

**FIGURE 3:**
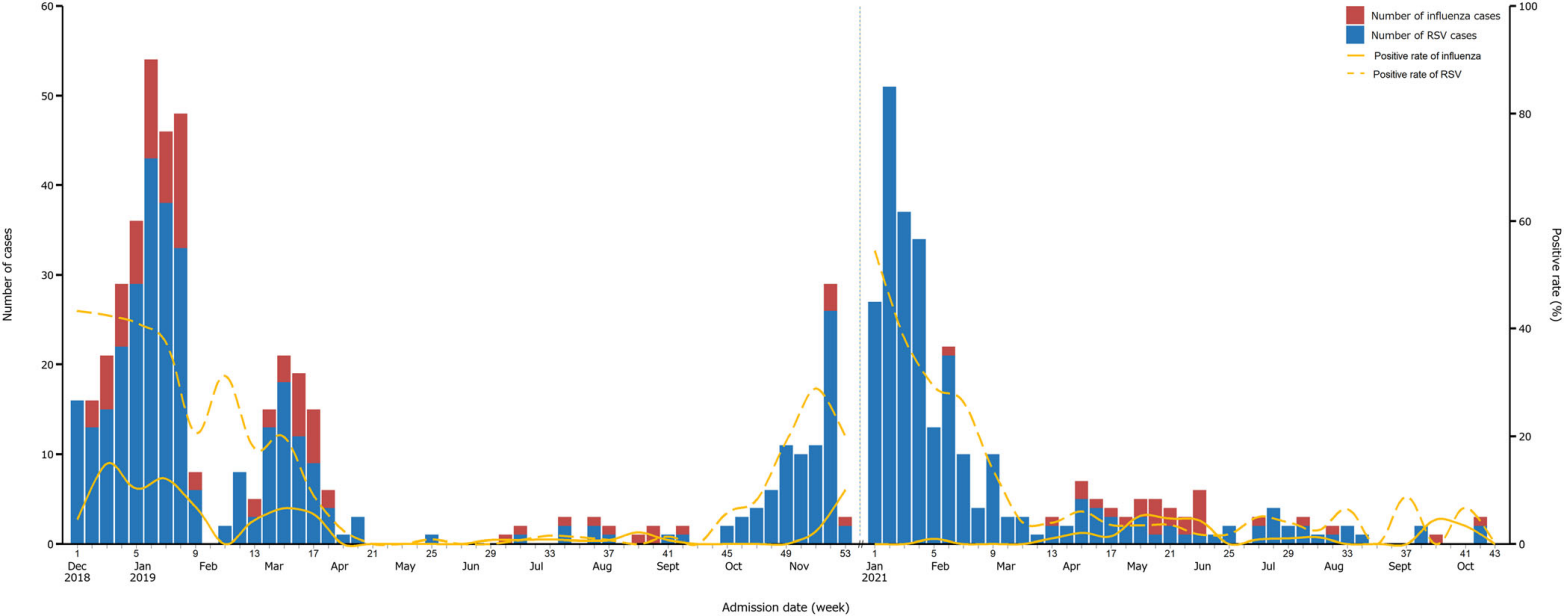
Temporal distribution of the numbers and positive rates of RSV and influenza‐infected patients in 2018–2019 and 2020–2021 study years. The vertical blue dashed line marked the separation of two study years. No. of RSV cases: 634 (372 in 2018–2019 and 262 in 2020–2021). No. of influenza cases: 121 (94 in 2018–2019 and 27 in 2020–2021).

The temporal distribution of RSV and influenza positive cases in both study years are shown in Figure 3. We determined, the annual epidemic season of RSV in 2018–2019 ended with March 2019. The next season started from mid‐October of 2019, with two consecutive weeks of RSV positivity per week exceeding the threshold of 10%.^16^ Although the RSV season of 2020–2021 ended earlier by the February 2021, than the March of previous study year, the RSV positivity rate after end of epidemic season was higher than that observed in April to September season of 2019 (Table S2). Influenza shared a similar epidemic season with RSV in 2018–2019. However, no usual peak was observed in the winter of 2020–2021.

### 3.3 Characteristics of RSV and influenza inpatients

The age distribution for patients with RSV or influenza infection varied between these study years (Table 1). The median age of children with RSV infection in 2018–2019 was lower than those from 2020–2021 (4.2 months, IQR: 2.3–12.6 vs. 7.2 months, IQR: 2.7–18.0, P < 0.05), so were the ones infected with influenza (27.3 months, IQR: 13.1–52.0 vs. 37.0 months, IQR: 12.0–82.5, P < 0.05). As compared with 2018–2019, fewer RSV‐infected patients in 2020–2021 were aged <6 months (58.9% vs. 43.9%), whereas the proportion of patients with RSV infection increased in the age group of 12 to <60 months. In patients with influenza, the maximum overall proportion of patients (30.6%) with an infection was observed in the group of 2‐ to 5‐year‐olds. This proportion decreased from 33.0% in 2018–2019 to 22.2% in 2020–2021. The median age of children infected with RSV was significantly younger than those infected with influenza in pooled study years (5.4 months, IQR: 2.4–15.3 vs. 29.1 months, IQR: 13.0–60.0, P < 0.05).

In addition to age changes, the distribution of local resident patients also changed during the pandemic. The proportion of patients living in Zhengzhou was higher in 2020–2021 than those from 2018–2019, in patients infected with RSV (82.1% vs. 65.1%, P < 0.05) as well as influenza (77.8% vs. 58.5%, P < 0.05) (Table 1).

RSV‐infected patients from 2020–2021 had a smaller proportion of patients with underlying conditions than the patients from 2018–2019 (7.6% [20/262] vs. 12.9% [48/372], P < 0.05). The most common underlying conditions included pre‐term birth (8.9% [33/372] in 2018–2019 and 6.5% [17/262] in 2020–2021) and congenital heart disease (CHD) (2.4% [9/372] in 2018–2019 and 0.8% [2/262] in 2020–2021). A similar decreasing trend of underlying conditions was observed in influenza‐infected patients (2018–2019: 13.8% [13/94] vs. 2020–2021: 7.4% [2/27]) (Table S3).

### 3.4 Clinical course and disease severity

As shown in Table 2, the median length of hospital stay among RSV patients was 6.0 days (IQR: 5.0–7.0) in both study years. Whereas patients with influenza had a longer hospital stay in 2018–2019 (6.0 days, IQR: 5.0–7.0) than 2020–2021 (5.0 days, IQR: 4.0–5.0, P < 0.05). The proportion of patients with pneumonia complications (diagnosed by radiographic findings or clinical signs, see Table 2) in RSV patients decreased slightly from 94.4% in 2018–2019 to 87.0% in 2020–2021, whereas it remarkably dropped to 33.3% from 71.3% (P < 0.05) (Table 2) in influenza‐infected patients. The clinical presentations and laboratory findings for RSV and influenza‐infected patients were generally similar before and during the pandemic (Tables S4 and S5).

**TABLE 2:** Clinical features of patients with RSV and influenza infection during hospitalization.

Characteristic	RSV‐infected patients			Influenza‐infected patients		
	Total	2018–2019	2020–2021	Total	2018–2019	2020–2021
	(n = 634)	(n = 372)	(n = 262)	(n = 121)	(n = 94)	(n = 27)
Interval from illness onset to admission, day, median (IQR)	4.0 (3.0–5.0)	4.0 (3.0–6.0)	4.0 (3.0–5.0)	5.0 (3.0–7.0)	5.5 (2.3–7.0)	4.0 (3.0–6.0)
Hospital length of stay, day, median (IQR)	6.0 (5.0–7.0)	6.0 (5.0–7.0)	6.0 (5.0–7.0)	5.0 (4.0–7.0)	6.0 (5.0–7.0)	5.0 (4.0–5.0)
Complications						
Pneumonia^a^, ^b^	579 (91.3)	351 (94.4)	228 (87.0)	76 (62.8)	67 (71.3)	9 (33.3)
Bronchitis	50 (7.9)	16 (4.3)	34 (13.0)	29 (24.0)	15 (16.0)	14 (51.9)
Hepatic function abnormality	26 (4.1)	25 (6.7)	1 (0.4)	4 (3.3)	3 (3.2)	1 (3.7)
Myocardial injury	16 (2.5)	15 (4.0)	1 (0.4)	2 (1.7)	2 (2.1)	0 (0)
Sepsis	10 (1.6)	6 (1.6)	4 (1.5)	7 (5.8)	7 (7.4)	0 (0)
Respiratory failure	19 (3.0)	15 (4.0)	4 (1.5)	8 (6.6)	8 (8.5)	0 (0)
Respiratory complication other than pneumonia and bronchitis^c^	19 (3.0)	3 (0.8)	16 (6.1)	2 (1.6)	2 (2.1)	0 (0)
Treatment						
Neuraminidase inhibitor treatment^d^	17 (2.7)	17 (4.6)	0 (0)	21 (17.4)	20 (21.3)	1 (3.7)
Other antiviral treatment^e^	6 (0.9)	4 (1.1)	2 (0.8)	1 (0.8)	1 (1.1)	0 (0)
Antibiotics treatment	610 (96.2)	356 (95.7)	254 (96.9)	115 (95.0)	90 (95.7)	25 (92.6)
Corticosteroid treatment	221 (34.9)	135 (36.3)	86 (32.8)	21 (17.4)	20 (21.3)	1 (3.7)
Supplementary oxygen treatment	144 (22.7)	99 (26.6)	45 (17.2)	19 (15.7)	19 (20.2)	0 (0)
Severe illness	33 (5.2)	28 (7.5)	5 (1.9)	15 (12.4)	15 (16.0)	0 (0)
Outcomes						
Respiratory support with non‐invasive positive pressure or invasive ventilation	21 (3.3)	18 (4.8)	3 (1.1)	7 (5.8)	7 (7.4)	0 (0)
ICU admission	32 (5.0)	27 (7.3)	5 (1.9)	15 (12.4)	15 (16.0)	0 (0)
In‐hospital death	1 (0.2)	1 (0.3)	0 (0)	0 (0)	0 (0)	0 (0)

*Note*: Figures are median (IQR) or numbers (%). Characteristics were compared in RSV/influenza positive patients between 2018–2019 and 2020–2021.

Abbreviations: ICU, intensive care unit; IQR, interquartile range.

^a^ In our study, pneumonia was diagnosed by physicians as children with clinical presentations of fever and/or respiratory symptoms with either condition as follows: (1) auscultation with fixed fine moist rale in the lungs; (2) radiographic findings of patchy shadowing or large area of consolidation.^30^

^b^ Bronchiolitis was usually diagnosed as pneumonia in this hospital.

^c^ Including laryngitis, laryngotracheitis, tracheitis, pertussis syndrome, amygdalitis, hydrothorax, aerothorax, and others.

^d^ Including oseltamivir and peramivir treatment.

^e^ Including interferon and ribavirin treatment.

The main treatment options for RSV infection relied on supportive care. The proportion of patients receiving any forms of oxygen treatment in 2018–2019 (26.6%) was more than those from 2020–2021 (17.2%, P < 0.05). The influenza‐infected patients received the well‐established antiviral treatment of neuraminidase inhibitors, and its proportion was higher in 2018–2019 than 2020–2021 (P < 0.05). Antibiotic treatment was often prescribed in both viral infections and their proportions did not differ during the pandemic. Overall, 96.2% (610/634) of RSV and 95.0% (115/121) influenza‐infected patients received antibiotics. 34.9% (221/634) RSV‐infected patients and 17.4% (21/121) influenza‐infected patients were treated with corticosteroids (P < 0.05) (Table 2).

7.5% (28/372) cases of RSV‐infected patients were identified as severe cases in 2018–2019 which dropped to 1.9% (5/262) in 2020–2021. The frequency of non‐invasive positive pressure or requirement of invasive ventilation was higher in 2018–2019 (4.8%) than in 2020–2021 (1.1%) (P < 0.05). Similar pattern was observed for the number of ICU admissions (7.3% vs. 1.9%, P < 0.05). The number of severe influenza cases decreased from 16.0% (15/94) in 2018–2019 to none in 2020–2021. Similarly, the number of ICU admissions significantly dropped from 16.0% (15/94) in 2018–2019 to 0% (0/27) in 2020–2021. In univariate and multivariate analyses, we found the children with CHD and living outside Zhengzhou were more likely to be associated with severe outcomes (Tables S6 and S7).

## 4 DISCUSSION

Our study reported a significantly reduced influenza activity in 2020–2021 which was similar to the studies from the United States and the United Kingdom.^4^, ^8^ However, no such impact of COVID‐19 was observed on the RSV activity in central China. In 2020–2021, the RSV positivity rate in children hospitalized with ARI remained high, in all groups of patients <5 years as compared to patients from 2018–2019. A study conducted in Hangzhou, China showed a high RSV positivity rate in children aged 1 month to 6 years during 2020 than the patients from 2019.^11^ Unlike studies from Australia, Brazil, the United States, or Europe,^17^, ^18^, ^19^, ^20^ the peak of RSV season was not delayed in our study. These changes in RSV and influenza activity patterns observed in our results were consistent with the surveillance data from 314 sentinel hospitals across mainland China since the September of 2020.^1^ And these results were similar to the studies from Guangdong and Zhejiang province of China.^10^, ^12^ This altered activity patterns between these viruses hinted that the public health measures against SARS‐CoV‐2 had more effective impact on influenza than RSV.^5^ One plausible reason for this observation could be the ability of RSV to survive for longer duration out of a host and its ability to quickly spread via direct contact.^9^, ^21^, ^22^ Additionally, the children probably could not fully benefit from current preventive measures like wearing masks, particularly those under 1 year, toward whom face masks were not recommended.

As compared to 2018–2019, a higher median age was observed in 2020–2021 in patients of ARI, RSV, and influenza (Table 1). During the pandemic, a similar older age structure of RSV and influenza patients was observed in the United States, France, and Australia.^5^, ^19^, ^20^ One possible explanation for these comparatively older age structures could be the “immunity debt,” resulting from the accumulation of immune‐naïve children due to strict implementation of NPIs in the first half of 2020. Children in early life are most vulnerable to RSV, influenza, and other respiratory viruses had a lesser chance of natural exposure to infection. Thus, the infection rates did not increase until the lockdown was lifted and these children were a little older.^7^, ^21^ Besides, a higher proportion of eligible ARI patients in 2020–2021 from the age groups of >5 months old led to the enrollment of a little older children which could be another reason for older RSV‐infected patients in our study (Table S1).

Meanwhile, the proportion of severe RSV or influenza illness was lower in 2020–2021 than 2018–2019. This finding was similar to the studies from Latin America, France, and Shanghai,^23^, ^24^, ^25^ where RSV or influenza‐related disease severity decreased after easing the NPIs. This changed severity of illness could be attributed to multiple factors. First, patients living outside Zhengzhou were found to be associated with an increased severity in our study, as the non‐local patients would travel to Zhengzhou when the illness became serious and looked for better medical resources. However, in 2020–2021, although the nationwide NPIs had been relaxed, the dropped mobility level possibly restricted to non‐local patients traveling to Zhengzhou, especially for children of early age (<6 months).^26^ Second, a large proportion of older children enrolled in 2020–2021 also contributed to a fewer number of ICU admissions or requirement of mechanical ventilation or in‐hospital death, as the younger age was associated with an increased severity of RSV and influenza infection.^27^, ^28^ Additionally, the lack of severe cases of influenza observed in 2020–2021 which could be mainly attributed to the suppression of influenza activity.

Several limitations to our study should be noted. First, we might have underestimated the influenza infection. For prevention of nosocomial infections, rapid antigen tests for influenza (Lateral Flow Immunoassay) (Wanfu Biotechnology Co., Ltd, Guangzhou, China; sensitivity: 52.47%, specificity: 99.41%) were constantly conducted in all outpatient departments of Henan Children's Hospital for patients with influenza‐like illness (ILI) (Table S8). For the patients who tested positive for influenza with an indication of hospitalization, pediatricians would provide a referral option for medical service in infectious diseases hospital, and only few patients were admitted to the infectious diseases ward in the month of peak epidemic. Since the diagnostic procedures for influenza were consistent before and during COVID‐19 pandemic, our results support the changed activity pattern of influenza which was consistent with surveillance data from China.^1^ Second, the enrolled ARI patients were little older in 2020–2021, which could bias the comparison of age structures of patients with RSV or influenza infection. However, eligible ARI patients in 2020–2021 with an increased age as compared to 2018–2019 showed, the study population indeed had an older age structure during the pandemic. Earlier studies showed, the young age of patients was associated with a higher positivity rate of RSV.^29^ The finding that an older age structure of enrolled patients and a considerably higher rate of RSV positivity in 2020–2021 than 2018–2019 strengthened the conclusion that, RSV infections remained at a higher level during the COVID‐19 pandemic.

## 5 CONCLUSIONS

The NPIs during COVID‐19 pandemic had varied impacts on common respiratory viruses. This was associated with a remarkably reduced incidence of influenza, while RSV infections remained at a higher level. This observation highlighted the need to find a most effective preventive strategy for RSV infections in childhood.

## AUTHOR CONTRIBUTIONS

**Lingshuang Ren:** Conceptualization; data curation; formal analysis; investigation; writing – original draft. **Li Lin:** Data curation; project administration. **Hua Zhang:** Data curation; project administration. **Qianli Wang:** Conceptualization; project administration; investigation; writing – original draft. **Yibing Cheng:** Project administration. **Qin Liu:** Project administration. **Bing Fang:** Investigation. **Linsen Xie:** Project administration. **Meng Wang:** Investigation. **Juan Yang:** Writing – review and editing. **Jinxin Guo:** Formal analysis. **Tianchen Zhang:** Investigation. **Hongkai Lian:** Supervision. **Jiangtao Wang:** Supervision. **Hongjie Yu:** Conceptualization; supervision; writing – review and editing.

## CONFLICT OF INTEREST

HY has received research funding from Sanofi and Shanghai Roche Pharmaceutical Company. All other authors declare no competing interests.

## ETHICS STATEMENT

The study was approved by School of Public Health, Fudan University (IRB No. 2018‐06‐0686 and IRB No. 2020‐12‐0864) and Research Ethics Commission of Henan Children's Hospital.

## PATIENT CONSENT

For patients under 7 years, informed consent forms were signed by their legal guardians. Patients aged between 7 and 11 years signed simplified forms, and their guardians signed the full versions. Patients older than 12 years and their guardians signed the full version forms.

### PEER REVIEW

The peer review history for this article is available at https://publons.com/publon/10.1111/irv.13103.

## Supporting information


**Table S1.** Demographic characteristics among all eligible, enrolled and excluded ARI patients
**Table S2.** Positive rate of RSV and influenza in enrolled ARI inpatients
**Table S3.** Detailed underlying conditions of patients before admission
**Table S4.** Clinical presentations of patients with RSV and influenza infection at admission
**Table S5.** Laboratory findings of patients infected with RSV and influenza infection at admission
**Table S6.** Univariate analysis of risk factors associated with ICU admission, mechanical ventilation or in‐hospital death
**Table S7.** Multivariate analyses of risk factors associated with ICU admission, mechanical ventilation or in‐hospital death
**Table S8.** Influenza rapid test of ILI outpatients

## Data Availability

The data are not publicly available due to privacy or ethical restrictions. De‐identified individual data that supports the findings of this study are available from the corresponding author on reasonable request. The data requestor will need to sign a data access agreement.
