# Supplementary material for: Epidemiological and clinical characteristics of respiratory syncytial virus and influenza infections in hospitalized children before and during the COVID‐19 pandemic in Central China
Source: Influenza Other Respir Viruses. 2023 Feb 2;17(2):e13103. doi: 10.1111/irv.13103 (PMC9895987; doi:10.1111/irv.13103)
Supplement: Supplementary file 1 — Table S1. Demographic characteristics among all eligible, enrolled and excluded ARI patients Table S2. Positive rate of RSV and influenza in enrolled ARI inpatients Table S3. Detailed underlying conditions of patients before admission Table S4. Clinical presentations of patients with RSV and influenza infection at admission Table S5. Laboratory findings of patients infected with RSV and influenza infection at admission Table S6. Univariate analysis of risk factors associated with ICU admission, mechanical ventilation or in‐hospital death Table S7. Multivariate analyses of risk factors associated with ICU admission, mechanical ventilation or in‐hospital death Table S8. Influenza rapid test of ILI outpatients [file IRV-17-e13103-s001.docx]

Supporting Information

Epidemiological and Clinical Characteristics of Respiratory Syncytial Virus and Influenza Infections in Hospitalized Children Before and During the COVID-19 Pandemic in Central China

Author list:^[[1]](#footnote-1)^

Lingshuang Ren^1^, Li Lin^2^, Hua Zhang^3^, Qianli Wang^4^, Yibing Cheng^2^, Qin Liu^3^, Bing Fang^2^, Linsen Xie^3^, Meng Wang^2^, Juan Yang^1^, Jinxin Guo^1^, Tianchen Zhang^5^, Hongkai Lian^3^, Jiangtao Wang^2^, Hongjie Yu^1, 4^

Affiliations:

1. School of Public Health, Fudan University, Key Laboratory of Public Health Safety, Ministry of Education, Shanghai, China

2. Children’s Hospital Affiliated to Zhengzhou University, Henan Children’s Hospital, Zhengzhou, China

3. Zhengzhou Central Hospital Affiliated to Zhengzhou University, Zhengzhou, China

4. Shanghai Institute of Infectious Disease and Biosecurity, Fudan University, Shanghai, China

5. Division of Infectious Disease, Jiangxi Province Center for Disease Control and Prevention, Nanchang, China

Correspondence

Prof. Hongjie Yu, School of Public Health, Fudan University, Key Laboratory of Public Health Safety, Ministry of Education, Shanghai, 200032, China. Tel: +86-21-54237628 Email: yhj@fudan.edu.cn

Contents

[1 Methods 3](#_Toc115812797)

[Laboratory detection of RSV and influenza infection 3](#_Toc115812798)

[Statistical analysis 3](#_Toc115812799)

[2 Results 3](#_Toc115812800)

[Characteristics of eligible ARI patients 3](#_Toc115812801)

[Clinical presentations, laboratory findings and treatment 4](#_Toc115812802)

[Factor associated with severe illness 4](#_Toc115812803)

[Influenza rapid test of ILI outpatients 4](#_Toc115812804)

[3 Table S1–S8 5](#_Toc115812805)

[4 References 17](#_Toc115812806)

# **1 Methods**

Laboratory detection of RSV and influenza infection

Collected specimens were stored at 4°C in a viral transport medium tube (Youkang Technology Co., Beijing, China) before processed. RNA was extracted from throat swab specimens using the QIAamp RNA mini kit (QIAGEN, Valencia, CA, USA) or an automatic viral nucleic acid extraction kit (Biogerm, Shanghai, China, Cat. No.: CZ-RE001-003) as per manufacturer’s instructions. Real Time-Polymerase Chain Reaction (RT-PCR) was performed using a panel of oligonucleotide primers and probes (Taqman^®^). The system used a TaqMan^®^ (oligonucleotide primers and probes) panel and a 20 µl reaction mixture containing 5 µl of TaqPath™ 1-step Multiplex Master Mix (Biogerm, Shanghai, China, Cat. No.: SJ-YH-001-3), 2.5µl of primer and probe solution, 5 µl of RNA template, and 7.5 µl of nuclease-free water. All collected samples were stored at 4°C and sent to the clinical labs by cold-chain transport for daily viral detection. In the study year 2018–2019, the viral detection was performed at Zhengzhou Central Hospital laboratory on ABI-7500 detection system (Applied Biosystems, CA, US). Whereas in 2020–2021, the detection was performed at Henan Children’s Hospital (East campus) on ABI-StepOneplus system (Applied Biosystems, CA, US).

The sequence of primers targeting RSV and influenza genes are as below:

RSV M gene

- Forward primer 5′-GGCAAATATGGAAACATACGTGAA-3′
- Reverse primer 5′-TCTTTTTCTAGGACATTGTAYTGAACAG-3′
- Probe 5′-FAM-CTGTGTATGTGGAGCCTTCGTGAAGCT-BHQ-3′

Influenza A M gene:

- Forward primer 5′-GACCRATCCTGTCACCTCTGAC-3′
- Reverse primer 5′-GGGCATTYTGGACAAAKCGTCTACG-3′
- Probe 5′-FAM-TGCAGTCCTCGCTCACTGGGCACG-TAMRA-3′

Influenza B NS gene:

- Forward primer 5′-TCCTCAACTCACTCTTCGAGCG-3′
- Reverse primer 5′-CGGTGCTCTTGACCAAATTGG-3′
- Probe 5′-FAM-CCAATTCGAGCAGCTGAAACTGCGGTG-TAMRA-3′

Primers and probes were selected as per published literature.^1^ The thermal cycling conditions were 25℃ for 2 min, 53℃ for 10 min, 95℃ for 2 min, and then 40 cycles of 95℃ for 3 s, and 60℃ for 30 s. In-house-prepared controls for Flu A, Flu B, and RSV RNA-infected controls, as well as no-template control were included in each run. An internal control targeting human ribonuclease protein (hRNP) gene was used to monitor the quality of collected samples.

Statistical analysis

We constructed multivariable logistic regression model to identify possible factors associated with severe illness, calculation of odds ratios (ORs) and 95% confidence intervals (95% CI). Candidate variables included age, sex, address, underlying conditions, types of viral infection, study year, and pharmaceutical treatment (neuraminidase inhibitors, antibiotics, and corticosteroids) before any clinical outcomes. A *p* value of <0.05 was considered statistically significant. All statistical analyses were performed in R v3.6.3.

# **2 Results**

Characteristics of eligible ARI patients

Among the 11218 admitted patients, there were 7093 eligible patients of acute respiratory infection (ARI). These eligible patients had a median age of 10.2 months (IQR: 2.8–34.0) and 60.0% of this population was male (Figure 1, Table S1). An increase in median age of eligible patients was observed in 2020–2021 (13.0 months, IQR: 3.4–39.0) as compared to patients from 2018–2019 (7.9 months, IQR: 2.6–30.2, *p*<0.05). Out of the eligible patients, 5250 (74.0%) with an informed consent were enrolled in the study and remaining 26.0% (1843/7093) patients were excluded. Another 11 patients were excluded due to incomplete medical records. In the end, 5239 patients remained in the study.

The demographic characteristics of 5239 enrolled and 1854 excluded patients are compared in Table S1. The median age of enrolled and excluded ARI patients in 2018–2019 was 8.0 and 7.6 months (*p*>0.05) resp., In 2020–2021, the median age of excluded patients (5.1 months, IQR: 2.3–20.0) was younger than enrolled patients (19.0 months, IQR: 6.4–43.0, *p*<0.05). Patients enrolled in study year 2018–2019 had a higher proportion of local residents (63.1%) than excluded (56.5%). Whereas the distribution of local residents was not significantly different between patients enrolled and excluded in study year 2020–2021.

Clinical presentations, laboratory findings and treatments

For patients infected with RSV or influenza, the most common symptoms after onset of illness were cough (RSV: 96.5%; influenza: 86.0%, *p*<0.05) and fever (RSV: 61.0%; influenza: 90.1%, *p*<0.05). As is shown in Table S4, the proportion of RSV-infected patients presenting moist crackles (73.9% vs. 65.6%) and dyspnea (5.6% vs. 1.1%) in 2018–2019 was significantly higher than those in 2020–2021 (*p*<0.05), but number of patients with tachypnea was lower (10.8% vs. 26.0%, *p*<0.05). For influenza-infected patients, wheezing in 2018–2019 was more common than in 2020–2021 (18.1% vs. 0%, *p*<0.05), whereas the proportion of patients with tachypnea (6.4% vs. 22.2%) was low.

In contrast to pneumonia, bronchitis in patients infected with RSV increased from 4.3% in 2018–2019 to 13.0% in 2020–2021. It also increased from 16.0% in 2018–2019 to 51.9% in 2020–2021 in patients infected with influenza (*p*<0.05). Complications in other systems, e.g., fewer hepatic function abnormalities and myocardial injuries for RSV infection and fewer sepsis for influenza infection were observed in 2020–2021 compared to 2018–2019 (Table 2).

Laboratory finding were stable before and during the pandemic, except for the hematology and biochemistry index, e.g., lactic dehydrogenase (LDH), creatine kinase-MB (CK-MB) for RSV infection, and neutrophils count and LDH for influenza. As compared to influenza, RSV-infected patients were found to have a higher levels of lymphocyte count and CD4:CD8 ratio in both study years. However, the levels of procalcitonin were significantly low (Table S5).

Factor associated with severe illness

For all the RSV or influenza-infected patients from both study years, the univariate analysis showed preterm birth, congenital heart disease (CHD), and low birth weight (LBW) were associated with a higher risk of severe illness (Table S6). RSV infection, corticosteroid and antibiotic treatment was appeared to be negatively associated with severe illness. In the final multivariable logistic model, we found children with CHD were at a higher risk than those without (aOR=43.0, 95% CI: 7.0–337.7). Antibiotics treatment during hospitalization showed a protective effect (aOR=0.01, 95% CI:0.01–0.03). Residing outside Zhengzhou was associated with severe outcomes (Table S7). The association of CHD with severe RSV or influenza infection was consistent with the other studies. ^2, 3^

Influenza rapid test of ILI outpatients

Table S8 showed the rapid influenza antigen test results by lateral flow immunoassays (LFIAs) for ILI patients in outpatient departments in East Campus of Henan Children’s Hospital during three consecutive epidemic seasons. The positivity rate for influenza considerably decreased in 2020–2021 season, with a rate of 0.3% for influenza A, as compared with 22.7% and 25.8% in 2018–2019 and 2019–2020 season respectively; and 0.2% for influenza B, compared with 1.7% and 11.4% in 2018–2019 and 2019–2020, respectively.

# **3 Table S1-S8**

Table S1. Demographic characteristics among all eligible, enrolled and excluded ARI patients

| **Characteristic** | **Total** | | |  | **2018–2019** | | |  | **2020–2021** | | |
| --- | --- | --- | --- | --- | --- | --- | --- | --- | --- | --- | --- |
|  | Overall | Enrolled | Excluded |  | Overall | Enrolled | Excluded |  | Overall | Enrolled | Excluded |
|  | (n=7093) | (n=5239) | (n=1854) |  | (n=4169) | (n=3275) | (n=894) |  | (n=2924) | (n=1964) | (n=960) |
| Age, month, median (IQR) | 10.2  (2.8–34.0) | 12.0  (3.0–37.2) | 6.2  (2.5–24.0) |  | 7.9  (2.6–30.2) | 8.0  (2.6–31.1) | 7.6  (2.8–27.8) |  | 13.0  (3.4–39.0) | 19.0  (6.4–43.0) | 5.1  (2.3–20.0) |
| Age group |  |  |  |  |  |  |  |  |  |  |  |
| 0–5 months | 2844 (40.1) | 1934 (36.9) | 910 (49.1) |  | 1864 (44.7) | 1461 (44.6) | 403 (45.1) |  | 980 (33.5) | 473 (24.1) | 507 (52.8) |
| 6–11 months | 941 (13.3) | 689 (13.2) | 252 (13.6) |  | 541 (13.0) | 430 (13.1) | 111 (12.4) |  | 400 (13.7) | 259 (13.2) | 141 (14.7) |
| 12–23 months | 1016 (14.3) | 791 (15.1) | 225 (12.1) |  | 560 (13.4) | 43 3 (13.2) | 127 (14.2) |  | 456 (15.6) | 358 (18.2) | 98 (10.2) |
| 24–59 months | 1607 (22.7) | 1282 (24.5) | 325 (17.5) |  | 847 (20.3) | 664 (20.3) | 183 (20.5) |  | 760 (26.0) | 618 (31.5) | 142 (14.8) |
| ≥ 5 years | 685 (9.7) | 543 (10.4) | 142 (7.7) |  | 357 (8.6) | 287 (8.8) | 70 (7.8) |  | 328 (11.2) | 256 (13.0) | 72 (7.5) |
| Male sex | 4257 (60.0) | 3136 (59.9) | 1121 (60.5) |  | 2515 (60.3) | 1982 (60.5) | 533 (59.6) |  | 1742 (59.6) | 1154 (58.8) | 588 (61.3) |
| Residence |  |  |  |  |  |  |  |  |  |  |  |
| Zhengzhou city | 4804 (67.7) | 3571 (68.2) | 1233 (66.5) |  | 2571 (61.7) | 2066 (63.1) | 505 (56.5) |  | 2233 (76.4) | 1505 (76.6) | 728 (75.8) |
| Henan Province outside Zhengzhou City | 2132 (30.1) | 1583 (30.2) | 549 (29.6) |  | 1485 (35.6) | 1144 (34.9) | 341 (38.1) |  | 647 (22.1) | 439 (22.4) | 208 (21.7) |
| Other areas outside Henan Province | 117 (1.6) | 83 (1.6) | 34 (1.8) |  | 90 (2.2) | 63 (1.9) | 27 (3.1) |  | 27 (0.9) | 20 (1.0) | 7 (0.7) |
| Missing data^†^ | 40 (0.6) | 2 (0.0) | 38 (2.0) |  | 23 (0.6) | 2 (0.1) | 21 (2.3) |  | 17 (0.6) | 0 (0) | 17 (1.8) |

Abbreviation: ARI, Acute respiratory infections; IQR, interquartile range.

Figures are numbers (%) unless stated otherwise. Characteristics were compared between enrolled and excluded ARI patients in 2018–2019 and 2020–2021, respectively.

^†^: Inpatients’ addresses were unfilled.

Table S2. Positive rate of RSV and influenza in enrolled ARI inpatients

| **Characteristics** | **RSV–infected patients** | | |  | **Influenza–infected patients** | | |
| --- | --- | --- | --- | --- | --- | --- | --- |
|  | Total | 2018–2019 | 2020–2021 |  | Total | 2018–2019 | 2020–2021 |
| Overall positive rate | 12.1 (634/5239) | 11.4 (372/3275) | 13.3 (262/1964) |  | 2.3 (121/5239) | 2.9 (94/3275) | 1.4 (27/1964) |
| Age group |  |  |  |  |  |  |  |
| 0–1 month | 16.4 (109/665) | 13.8 (71/516) | 25.5 (38/149) |  | 0.4 (3/671) | 0.6 (3/516) | 0 (0/155) |
| 2–3 months | 17.8 (169/952) | 15.7 (112/714) | 23.9 (57/238) |  | 0.8 (8/947) | 1.0 (7/714) | 0.4 (1/233) |
| 4–5 months | 17.7 (56/317) | 15.6 (36/231) | 23.3 (20/86) |  | 0.9 (3/316) | 0.4 (1/231) | 2.4 (2/85) |
| 6–11 months | 13.4 (92/689) | 12.8 (55/430) | 14.3 (37/259) |  | 2.3 (16/689) | 2.8 (12/430) | 1.5 (4/259) |
| 12–23 months | 14.8 (117/791) | 12.7 (55/433) | 17.3 (62/358) |  | 2.9 (23/791) | 4.8 (21/433) | 0.6 (2/358) |
| 24–59 months | 6.6 (85/1282) | 5.7 (38/664) | 7.6 (47/618) |  | 2.9 (37/1282) | 4.7 (31/664) | 1.0 (6/618) |
| ≥ 5 years | 1.1 (6/543) | 1.7 (5/287) | 0.4 (1/256) |  | 5.7 (31/543) | 6.6 (19/287) | 4.7 (12/256) |
| Month of admission |  |  |  |  |  |  |  |
| December of the former year | 46.2 (103/223) | 42.9 (76/177) | 58.7 (27/46) |  | 8.1 (18/223) | 10.2 (18/177) | 0 (0/46) |
| January of the latter year | 37.5 (285/761) | 35.8 (139/388) | 39.1 (146/373) |  | 5.5 (42/761) | 10.6 (41/388) | 0.3 (1/373) |
| February | 23.4 (49/209) | 24.1 (13/54) | 23.2 (36/155) |  | 1.0 (2/209) | 3.7 (2/54) | 0 (0/155) |
| March | 11.7 (61/523) | 16.8 (52/310) | 4.2 (9/213) |  | 4.0 (21/523) | 6.5 (20/310) | 0.5 (1/213) |
| April | 3.5 (22/637) | 2.4 (8/331) | 4.6 (14/306) |  | 0.8 (5/637) | 0 (0/331) | 1.6 (5/306) |
| May | 1.7 (9/538) | 0.3 (1/310) | 3.5 (8/228) |  | 2.4 (13/538) | 0 (0/310) | 5.7 (13/228) |
| June | 0.9 (5/587) | 0 (0/334) | 2.0 (5/253) |  | 0.7 (4/587) | 0.3 (1/334) | 1.2 (3/253) |
| July | 2.3 (12/515) | 1.0 (3/295) | 4.1 (9/220) |  | 0.6 (3/515) | 0.7 (2/295) | 0.5 (1/220) |
| August | 1.7 (7/424) | 0.9 (3/335) | 4.5 (4/89) |  | 0.9 (4/424) | 0.9 (3/335) | 1.1 (1/89) |
| September | 1.2 (4/332) | 0.7 (2/284) | 4.2 (2/48) |  | 1.2 (4/332) | 1.1 (3/284) | 2.1 (1/48) |
| October | 6.0 (14/233) | 6.0 (12/200) | 6.1 (2/33) |  | 0.4 (1/233) | 0 (0/200) | 3.0 (1/33) |
| November | 24.5 (63/257) | 24.5 (63/257) | – |  | 1.6 (4/257) | 1.6 (4/257) | – |

Abbreviation: RSV, respiratory syncytial virus.

Figures are % (n/N) where the denominators denote the corresponding number of ARI patients enrolled unless stated otherwise. Study year 2018–2019 began on December 1, 2018 and ended on November 30, 2019; study year 2020–2021 began on December 29, 2020 and ended on October 20, 2021.

Table S3. Detailed underlying conditions of patients before admission

| **Characteristic** | **Enrolled ARI patients** | | |  | **RSV-infected patients** | | |  | **Influenza-infected patients** | | |
| --- | --- | --- | --- | --- | --- | --- | --- | --- | --- | --- | --- |
|  | Total | 2018–2019 | 2020–2021 |  | Total | 2018–2019 | 2020–2021 |  | Total | 2018–2019 | 2020–2021 |
|  | (n=5239) | (n=3275) | (n=1964) |  | (n =634) | (n =372) | (n =262) |  | (n =121) | (n =94) | (n =27) |
| Underlying conditions |  |  |  |  |  |  |  |  |  |  |  |
| Preterm birth | 345 (6.6) | 240 (7.3) | 105 (5.3) |  | 50 (7.9) | 33 (8.9) | 17 (6.5) |  | 10 (8.3) | 9 (9.6) | 1 (3.7) |
| Congenital heart disease | 146 (2.8) | 121 (3.7) | 25 (1.3) |  | 11 (1.7) | 9 (2.4) | 2 (0.8) |  | 1 (0.8) | 1 (1.1) | 0 (0) |
| Metabolic diseases^†^ | 16 (0.3) | 16 (0.5) | 0 (0) |  | 2 (0.3) | 2 (0.5) | 0 (0) |  | 2 (1.7) | 2 (2.1) | 0 (0) |
| Airway dysplasia | 36 (0.7) | 35 (1.1) | 1 (0.1) |  | 2 (0.3) | 2 (0.5) | 0 (0) |  | 1 (0.8) | 1 (1.1) | 0 (0) |
| Asthma | 17 (0.3) | 11 (0.3) | 6 (0.3) |  | 0 (0) | 0 (0) | 0 (0) |  | 2 (1.7) | 2 (2.1) | 0 (0) |
| Other^‡^ | 199 (3.8) | 166 (5.1) | 33 (1.7) |  | 14 (2.2) | 11 (3.0) | 3 (1.1) |  | 6 (5.0) | 5 (5.3) | 1 (3.7) |
| Gestational age of preterm infants |  |  |  |  |  |  |  |  |  |  |  |
| <29 weeks | 25/345 (7.2) | 17/240 (7.1) | 8/105 (7.6) |  | 4/50 (8.0) | 4/33 (12.1) | 0/17 (0, 0) |  | 1/10 (10.0) | 1/9 (11.1) | 0/1 (0) |
| 29–32 weeks | 28/345 (8.1) | 21/240 (8.8) | 7/105 (6.7) |  | 2/50 (4.0) | 2/33 (6.1) | 0/17 (0) |  | 2/10 (20.0) | 2/9 (22.2) | 0/1 (0) |
| 32–36 weeks | 133/345 (38.6) | 87/240 (36.3) | 46/105 (43.8) |  | 24/50 (48.0) | 13/33 (39.4) | 11/17 (64.7) |  | 4/10 (40.0) | 3/9 (33.3) | 1/1 (100.0) |
| >=36 weeks | 159/345 (46.1) | 115/240 (47.9) | 44/105 (41.9) |  | 20/50 (40.0) | 14/33 (42.4) | 6/17 (35.3) |  | 3/10 (30.0) | 3/9 (33.3) | 0/1 (0) |
| Birth weight |  |  |  |  |  |  |  |  |  |  |  |
| LBW | 338 (6.5) | 260 (7.9) | 78 (4.0) |  | 32 (5.0) | 19 (5.1) | 13 (5.0) |  | 9 (6.4) | 9 (8.0) | 0 (0) |
| Normal | 4461 (85.1) | 2714 (82.9) | 1747 (89.0) |  | 549 (86.6) | 312 (83.9) | 237 (90.5) |  | 96 (68.6) | 69 (61.1) | 27 (100.0) |
| HBW | 412 (7.9) | 299 (9.1) | 113 (5.8) |  | 46 (7.3) | 34 (9.1) | 12 (4.6) |  | 10 (7.1) | 10 (8.8) | 0 (0) |
| Missing data^§^ | 28 (0.5) | 2 (0.1) | 26 (1.3) |  | 7 (1.1) | 7 (1.9) | 0 (0) |  | 6 (4.3) | 6 (5.3) | 0 (0) |

Abbreviations: ARI, Acute respiratory infections; RSV, respiratory syncytial virus; LBW: low birth weight, born weighting less than 2500g; HBW: high birth weight, born weighting more than 4000g.

Figures are numbers (%) unless stated otherwise. Characteristics were compared in RSV/influenza positive patients between 2018–2019 and 2020–2021.

^†^Include congenital hypothyroidism, congenital adrenal hyperplasia, galactosemia, phenylketonuria, G6PD deficiency, primary carnitine deficiency and methylmalonic acidemia.

^‡^Include hepatic disease, valvular disease, myocardial damage, cardiac insufficiency, Renpenning Syndrome, Down's syndrome, congenital gastrointestinal disease, and Kawasaki Disease.

^§^Inpatients’ birth weight were unfilled.

Table S4. Clinical presentations of patients with RSV and influenza infection at admission

| **Characteristic** | **RSV-infected inpatients** | | |  | **Influenza-infected inpatients** | | |
| --- | --- | --- | --- | --- | --- | --- | --- |
|  | Total | 2018–2019 | 2020–2021 |  | Total | 2018–2019 | 2020–2021 |
|  | (n =634) | (n =372) | (n =262) |  | (n =121) | (n =94) | (n =27) |
| Clinical symptoms |  |  |  |  |  |  |  |
| Cough | 612 (96.5) | 361 (97.0) | 251 (95.8) |  | 104 (86.0) | 84 (89.4) | 20 (74.1) |
| Sputum | 497 (78.4) | 295 (79.3) | 202 (77.1) |  | 65 (53.7) | 49 (52.1) | 16 (59.3) |
| Fever | 387 (61.0) | 209 (56.2) | 178 (67.9) |  | 109 (90.1) | 84 (89.4) | 25 (92.6) |
| Rhinorrhea | 249 (39.3) | 144 (38.7) | 105 (40.1) |  | 51 (42.1) | 37 (39.4) | 14 (51.9) |
| Wheezing | 232 (36.6) | 141 (37.9) | 91 (34.7) |  | 17 (14.0) | 17 (18.1) | 0 (0) |
| Poor sleeping | 151 (23.8) | 84 (22.6) | 67 (25.6) |  | 31 (25.6) | 27 (28.7) | 4 (14.8) |
| Vomiting | 113 (17.8) | 66 (17.7) | 47 (17.9) |  | 23 (19.0) | 21 (22.3) | 2 (7.4) |
| Sneeze | 66 (10.4) | 48 (12.9) | 18 (6.9) |  | 11 (9.1) | 8 (8.5) | 3 (11.1) |
| Tachypnea | 108 (17.0) | 40 (10.8) | 68 (26.0) |  | 12 (9.9) | 6 (6.4) | 6 (22.2) |
| Diarrhea | 70 (11.0) | 32 (8.6) | 38 (14.5) |  | 13 (10.7) | 12 (12.8) | 1 (3.7) |
| Dyspnea | 24 (3.8) | 21 (5.6) | 3 (1.1) |  | 8 (6.6) | 8 (8.5) | 0 (0) |
| Cyanosis | 14 (2.2) | 10 (2.7) | 4 (1.5) |  | 9 (7.4) | 9 (9.6) | 0 (0) |
| Chills | 17 (2.7) | 8 (2.2) | 9 (3.4) |  | 11 (9.1) | 11 (11.7) | 0 (0) |
| Drowsiness | 4 (0.6) | 3 (0.8) | 1 (0.4) |  | 5 (4.1) | 5 (5.3) | 0 (0) |
| Headache | 4 (0.6) | 3 (0.8) | 1 (0.4) |  | 5 (4.1) | 4 (4.3) | 1 (3.7) |
| Nausea | 7 (1.1) | 2 (0.5) | 5 (1.9) |  | 4 (3.3) | 4 (4.3) | 0 (0) |
| Dizziness | 1 (0.2) | 1 (0.3) | 0 (0) |  | 3 (2.5) | 3 (3.2) | 0 (0) |
| Myalgia | 1 (0.2) | 0 (0) | 1 (0.4) |  | 1 (0.8) | 1 (1.1) | 0 (0) |
| Malaise | 0 (0) | 0 (0) | 0 (0) |  | 8 (6.6) | 6 (6.4) | 2 (7.4) |
| Consciousness disturbance | 0 (0) | 0 (0) | 0 (0) |  | 3 (2.5) | 3 (3.2) | 0 (0) |
| Sore throat | 0 (0) | 0 (0) | 0 (0) |  | 2 (1.7) | 2 (2.1) | 0 (0) |
| Signs |  |  |  |  |  |  |  |
| Pharyngeal congestion | 496 (78.2) | 314 (84.4) | 182 (69.5) |  | 107 (88.4) | 83 (88.3) | 24 (88.9) |
| Moist crackles | 447 (70.5) | 275 (73.9) | 172 (65.6) |  | 71 (58.7) | 58 (61.7) | 13 (48.1) |
| Rhonchi | 174 (27.4) | 106 (28.5) | 68 (26.0) |  | 17 (14.0) | 16 (17.0) | 1 (3.7) |
| Swollen tonsils | 76 (12.0) | 31 (8.3) | 45 (17.2) |  | 43 (35.5) | 31 (33.0) | 12 (44.4) |
| Rash | 10 (1.6) | 5 (1.3) | 5 (1.9) |  | 1 (0.8) | 1 (1.1) | 0 (0) |

Abbreviations: RSV, respiratory syncytial virus.

Figures are numbers (%) unless stated otherwise. Characteristics were compared in RSV or influenza positive patients between 2018–2019 and 2020–2021.

Table S5. Laboratory findings of patients infected with RSV and influenza infection at admission

| **Characteristics** | **RSV-infected patients** | | |  | **Influenza-infected patients** | | |
| --- | --- | --- | --- | --- | --- | --- | --- |
|  | Total | 2018–2019 | 2020–2021 |  | Total | 2018–2019 | 2020–2021 |
|  | (n =634) | (n =372) | (n =262) |  | (n =121) | (n =94) | (n =27) |
| Hematology^†^ |  |  |  |  |  |  |  |
| White blood cell, × 10⁹/L | 8.1 (6.4–10.2) | 8.1 (6.6–10.4) | 8.2 (6.2–10.1) |  | 6.3 (4.6–9.2) | 6.9 (4.8–10.0) | 5.3 (4.1–6.0) |
| <5 | 42/569 (7.4) | 22/322 (6.8) | 20/247 (8.1) |  | 33/108 (30.6) | 23/86 (26.7) | 10/22 (45.5) |
| 5–12 | 450/569 (79.1) | 252/322 (78.3) | 198/247 (80.2) |  | 59/108 (54.6) | 49/86 (57.0) | 10/22 (45.5) |
| >12 | 77/569 (13.5) | 48/322 (14.9) | 29/247 (11.7) |  | 16/108 (14.8) | 14/86 (16.3) | 2/22 (9.1) |
| Neutrophils, × 10⁹/L | 2.3 (1.5–3.5) | 2.31 (1.6–3.8) | 2.1 (1.5–3.3) |  | 2.4 (1.4–4.5) | 2.6 (1.4–4.9) | 1.7 (1.0–2.1) |
| <2 | 238/569 (41.8) | 128/322 (39.8) | 110/247 (44.5) |  | 45/108 (41.7) | 30/86 (34.9) | 15/22 (68.2) |
| 2–7.2 | 295/569 (51.8) | 168/322 (52.2) | 127/247 (51.4) |  | 47/108 (43.5) | 41/86 (47.7) | 6/22 (27.3) |
| >7.2 | 36/569 (6.3) | 26/322 (8.1) | 10/247 (4.0) |  | 16/108 (14.8) | 15/86 (17.4) | 1/22 (4.5) |
| Lymphocyte, × 10⁹/L | 4.8 (3.4–6.2) | 4.8 (3.4–6.2) | 4.8 (3.5–6.1) |  | 3.0 (1.7–4.2) | 3.00 (1.7–4.2) | 3.0 (2.3–4.1) |
| <1.2 | 7/569 (1.2) | 6/322 (1.9) | 1/247 (0.4) |  | 12/108 (11.1) | 10/86 (11.6) | 2/22 (9.1) |
| 1.2–4.8 | 283/569 (49.7) | 159/322 (49.4) | 124/247 (50.2) |  | 77/108 (71.3) | 59/86 (68.6) | 18/22 (81.8) |
| >4.8 | 279/569 (49.0) | 157/322 (48.8) | 122/247 (49.4) |  | 19/108 (17.6) | 17/86 (19.8) | 2/22 (9.1) |
| Hemoglobin, g/L | 110.0 (101.0–118.0) | 108.0 (99.0–115.0) | 112.0 (104.5–121.0) |  | 114.0 (105.0–122.0) | 111.5 (105.0–120.0) | 118.0 (106.0–125.0) |
| Platelet, × 10⁹/L | 388.0 (295.0–493.0) | 389.5 (295.0–489.5) | 388.0 (296.5–495.5) |  | 267.0 (213.5–339.5) | 269.5 (217.8–359.8) | 242.0 (179.0–305.0) |
| Serum biochemistry^‡^ |  |  |  |  |  |  |  |
| Alanine aminotransferase, U/L | 33.1 (16.5–48.0) | 37.2 (18.6–50.3) | 27.9 (15.6–44.2) |  | 18.1 (12.2–36.5) | 18.5 (11.7–37.5) | 15.8 (13.5–30.1) |
| ≤50 | 457/595 (76.8) | 268/360 (74.4) | 189/235 (80.4) |  | 100/115 (87.0) | 76/89 (85.4) | 24/26 (92.3) |
| >50 | 138/595 (23.2) | 92/360 (25.6) | 46/235 (19.6) |  | 15/115 (13.0) | 13/89 (14.6) | 2/26 (7.7) |
| Aspartate aminotransferase, U/L | 40.10 (33.2–49.2) | 38.6 (31.9–48.6) | 41.7 (35.1–50.3) |  | 37.6 (28.8–48.9) | 37.6 (28.8–48.9) | 36.5 (32.3–49.3) |
| ≤60 | 522/598 (87.3) | 317/362 (87.6) | 205/236 (86.9) |  | 102/115 (88.7) | 79/89 (88.8) | 23/26 (88.5) |
| >60 | 76/598 (12.7) | 45/362 (12.4) | 31/236 (13.1) |  | 13/115 (11.3) | 10/89 (11.2) | 3/26 (11.5) |
| Lactic dehydrogenase, U/L | 314.0 (274.7–369.0) | 302.1 (268.4–357.1) | 330.0 (284.2–381.8) |  | 340.0 (285.5–389.8) | 340.0 (285.5–389.8) | 312.6 (258.2–352.2) |
| ≤290 | 213/593 (35.9) | 148/359 (41.2) | 65/234 (27.8) |  | 33/113 (29.2) | 23/87 (26.4) | 10/26 (38.5) |
| >290 | 380/593 (64.1) | 211/359 (58.8) | 169/234 (72.2) |  | 80/113 (70.8) | 64/87 (73.6) | 16/26 (61.5) |
| Serum creatinine, μmol/L | 21.6 (16.8–26.6) | 24.20 (18.5–29.40) | 19.0 (15.0–23.0) |  | 28.7 (24.1–36.0) | 28.9 (24.7–36.0) | 26.2 (23.7–32.8) |
| ≤65 | 554/554 (100.0) | 333/333 (100) | 221/221 (100.0) |  | 110/114 (96.5) | 87/89 (97.8) | 23/25 (92.0) |
| >65 | 0/554 (0) | 0/333 (0) | 0/221 (0) |  | 4/114 (3.5) | 2/89 (2.2) | 2/25 (8.0) |
| Creatine kinase, U/L | 77.0 (53.0–106.7) | 76.3 (52.2–106.1) | 77.1 (54.3–107.8) |  | 76.0 (53.0–115.7) | 76.0 (52.0–110.5) | 75.7 (57.8–126.4) |
| ≤200 | 566/593 (95.4) | 341/359 (95.0) | 225/234 (96.2) |  | 103/113 (91.2) | 80/87 (92.0) | 23/26 (88.5) |
| >200 | 27/593 (4.6) | 18/359 (5.0) | 9/234 (3.8) |  | 10/113 (8.8) | 7/87 (8.0) | 3/26 (11.5) |
| Creatine kinase–MB, U/L | 23.0 (17.7–29.2) | 22.0 (16.0–28.5) | 25.0 (19.9–30.0) |  | 20.0 (15.0–26.0) | 21.0 (16.0–26.8) | 18.4 (15.0–24.8) |
| ≤25 | 351/595 (59.0) | 226/361 (62.6) | 125/234 (53.4) |  | 82/115 (71.3) | 62/89 (69.7) | 20/26 (76.9) |
| >25 | 244/595 (41.0) | 135/361 (37.4) | 109/234 (46.6) |  | 33/115 (28.7) | 27/89 (30.3) | 6/26 (23.1) |
| Infection or immunological marker^§^ |  |  |  |  |  |  |  |
| C–reactive protein, mg/L | 4.6 (1.5–13.1) | 6.1 (1.7–15.6) | 3.0 (1.3–7.6) |  | 7.6 (2.4–27.5) | 11.0 (3.6–34.3) | 2.4 (1.3–5.3) |
| ≤10 | 162/223 (72.6) | 100/154 (64.9) | 62/69 (89.9) |  | 31/61 (50.8) | 25/54 (46.3) | 6/7 (85.7) |
| >10 | 71/223 (31.8) | 54/154 (35.1) | 17/69 (24.6) |  | 30/61 (49.2) | 29/54 (53.7) | 1/7 (14.3) |
| Procalcitonin, ng/mL | 0.1 (0.1–0.1) | 0.1 (0.1–0.1) | 0.1 (0.1–0.1) |  | 0.2 (0.1–0.2) | 0.2 (0.1–0.3) | 0.1 (0.1–0.2) |
| ≤0.1 | 345/607 (56.8) | 201/364 (55.2) | 144/243 (59.3) |  | 35/111 (31.5) | 25/86 (29.1) | 10/25 (40.0) |
| >0.1 | 262/607 (43.2) | 163/364 (44.8) | 99/243 (40.7) |  | 76/111 (68.5) | 61/86 (70.9) | 15/25 (60.0) |
| Erythrocyte sedimentation, mm/hour | 15.0 (7.0–27.0) | 11.0 (4.5–19.0) | 21.0 (11.0–38.0) |  | 16.0 (8.0–27.0) | 16.5 (8.0–27.8) | 11.5 (6.0–23.3) |
| ≤20 | 239/364 (65.7) | 165/215 (76.7) | 74/149 (49.7) |  | 65/108 (60.2) | 48/84 (57.1) | 17/24 (70.8) |
| >20 | 125/364 (34.3) | 50/215 (23.3) | 75/149 (50.3) |  | 43/108 (39.8) | 36/84 (42.9) | 7/24 (29.2) |
| Interleukin–6, pg/mL | 20.7 (8.8–73.3) | 20.9 (7.3–54.5) | 20.6 (9.7–80.2) |  | 19.3 (8.6–95.8) | 22.7 (11.2–251.2) | 17.2 (8.1–55.6) |
| ≤7.0 | 61/336 (18.2) | 25/105 (23.8) | 36/231 (15.6) |  | 14/46 (30.4) | 5/22 (22.7) | 9/24 (37.5) |
| >7 | 275/336 (81.8) | 80/105 (76.2) | 195/231 (84.4) |  | 32/46 (69.6) | 17/22 (77.3) | 15/24 (62.5) |
| CD4+: CD8+ ratio | 2.4 (1.7–3.2) | 2.5 (1.7–3.4) | 2.3 (1.7–3.0) |  | 1.5 (1.1–1.9) | 1.5 (1.1–2.1) | 1.4 (1.0–1.8) |
| ≤2.0 | 181/498 (36.3) | 96/271 (35.4) | 85/227 (37.4) |  | 62/81 (76.5) | 41/57 (71.9) | 21/24 (87.5) |
| >2 | 317/498 (63.7) | 175/271 (64.6) | 142/227 (62.6) |  | 19/81 (23.5) | 16/57 (28.1) | 3/24 (12.5) |

Abbreviations: RSV, respiratory syncytial virus.

Figures are the median (IQR) or n/N (%), where the denominators represent the numbers of patients with corresponding laboratory results. Characteristics were compared in RSV or influenza positive patients between 2018–2019 and 2020–2021.

^†^Normal range: white blood cell, 5.0–12.0×10⁹/L; neutrophils count, 1.2–7.2×10⁹/L; lymphocyte count, 1.2–4.8×10⁹/L; hemoglobin, 105–145 g/L; platelet count, 140–440×10⁹/L.

^‡^Normal range: alanine aminotransferase, ≤50 U/L; aspartate aminotransferase, ≤60 U/L; lactic dehydrogenase, ≤290 U/L; Serum creatinine, ≤65 μmol/L; Blood urea nitrogen, ≤7.6 mmol/L; creatine kinase, ≤200 U/L; creatine kinase-MB, ≤25 U/L.

^§^Normal range: C-reactive protein, ≤10 mg/L; procalcitonin, 0–0.25 ng/mL; erythrocyte sedimentation rate, 0–20 mm/hour; interleukin-6, ≤7 pg/mL; CD4+: CD8+ ratio, ≤2.0.

Table S6. Univariate analysis of risk factors associated with ICU admission, mechanical ventilation or in-hospital death

| **Subgroups** | **n (%)** | **Univariate analysis** | |
| --- | --- | --- | --- |
|  |  | OR (95% CI) | *p* value |
| Sex |  |  |  |
| Male | 451 (59.7) | Reference |  |
| Female | 304 (40.3) | 1.7 (0.9–3.0) | 0.087* |
| Residence |  |  |  |
| Zhengzhou City | 533 (70.6) | Reference |  |
| Outside Zhengzhou City | 220 (29.1) | 10.6 (5.4–22.9) | <0.001 |
| Unknown | 2 (0.3) | 52.3 (2.0–1387.9) | 0.006 |
| Age group |  |  |  |
| 0–5 months | 348 (46.1) | Reference |  |
| 6–11 months | 108 (14.3) | 1.4 (0.6–3.2) | 0.455 |
| 12–23 months | 140 (18.5) | 1.5 (0.7–3.1) | 0.321 |
| 24–59 months | 122 (16.2) | 1.1 (0.4–2.5) | 0.908 |
| ≥5 years | 37 (4.9) | 1.5 (0.3–4.8) | 0.512 |
| Preterm birth |  |  |  |
| No | 695 (92.1) | Reference |  |
| Yes | 60 (7.9) | 3.0 (1.3–6.2) | 0.006 |
| Congenital heart disease |  |  |  |
| No | 743 (98.4) | Reference |  |
| Yes | 12 (1.6) | 54.2 (15.5–251.5) | <0.001 |
| Low birth weight |  |  |  |
| No | 703 (93.1) | Reference |  |
| Yes | 52 (6.9) | 5.6 (2.6–11.3) | <0.001 |
| History of pneumonia |  |  |  |
| No | 667 (88.3) | Reference |  |
| Yes | 88 (11.7) | 1.8 (0.8–3.8) | 0.118 |
| Viral infection |  |  |  |
| Influenza | 121 (16.0) | Reference |  |
| RSV | 634 (84.0) | 0.4 (0.2–0.8) | 0.004 |
| Study year |  |  |  |
| 2018–2019 | 466 (61.7) | Reference |  |
| 2020–2021 | 289 (38.3) | 0.2 (0.1–0.4) | <0.001 |
| Neuraminidase inhibitor treatment |  |  |  |
| Not received | 738 (97.7) | Reference |  |
| Received | 17 (2.3) | 2.0 (0.3–7.4) | 0.365 |
| Corticosteroid treatment |  |  |  |
| Not received | 544 (72.1) | Reference |  |
| Received | 211 (27.9) | 0.2 (0.1–0.6) | 0.006 |
| Antibiotic treatment |  |  |  |
| Not received | 60 (7.9) | Reference |  |
| Received | 695 (92.1) | 0.01 (0.01–0.03) | <0.001 |

Abbreviations: ICU, intensive care unit; OR, odds ratio; CI, confidence interval; RSV, respiratory syncytial virus.

755 subjects detected with RSV or influenza positive in two years were included into analysis: 372 RSV and 94 influenza-infected patients in 2018–2019, and 262 RSV and 27 influenza-infected patients in 2020–2021.

*The underlined *p*-value was >0.05 but <0.1.

Table S7. Multivariate analyses of risk factors associated with ICU admission, mechanical ventilation or in-hospital death

| **Subgroup** | **n (%)** | **Multivariate analysis** | |
| --- | --- | --- | --- |
|  |  | aOR (95% CI) | *p* value |
| Residence |  |  |  |
| Zhengzhou City | 533 (70.6) | Reference |  |
| Outside Zhengzhou City | 220 (29.1) | 11.7 (4.6–33.8) | <0.001 |
| Unknown | 2 (0.3) | 29.9 (0.4–2202.8) | 0.154 |
| Congenital heart disease |  |  |  |
| No | 743 (98.4) | Reference |  |
| Yes | 12 (1.6) | 43.0 (7.0–337.7) | <0.001 |
| Antibiotics treatment during hospitalization |  |  |  |
| Not received | 60 (7.9) | Reference |  |
| Received | 695 (92.1) | 0.01 (0.01–0.03) | <0.001 |

Abbreviations: ICU, intensive care unit; aOR, adjusted odds ratio; CI, confidence interval.

755 subjects detected RSV or influenza positive in two years were included into analysis: 372 RSV and 94 influenza-infected patients in 2018–2019, and 262 RSV and 27 influenza-infected patients in 2020–2021.

Table S8. Influenza rapid test of ILI outpatients

| **Characteristics** | **November–April in 2018–2019** | **November–April in 2019–2020** | **November–March 2020–2021^†^** |
| --- | --- | --- | --- |
| Number of episodes undertaken |  |  |  |
| Influenza A antigen | 72630 | 56469 | 3114 |
| Influenza B antigen | 72624 | 56462 | 3114 |
| Number of positive results |  |  |  |
| Influenza A antigen | 18206 | 12827 | 8 |
| Influenza B antigen | 8723 | 954 | 6 |
| Positive rate (%) |  |  |  |
| Influenza A antigen | 25.1 | 22.7 | 0.3 |
| Influenza B antigen | 11.4 | 1.7 | 0.2 |

Abbreviations: ILI, influenza-like illness.

^†^Data was obtained up to March 4, 2021

# **4 References**

1. Ali SA, Gern JE, Hartert TV, Edwards KM, Griffin MR, Miller EK, et al. Real-world comparison of two molecular methods for detection of respiratory viruses. *Virol J*. 2011; 8: 332. https://doi.org/10.1186/1743-422X-8-332.
2. Aikphaibul P, Theerawit T, Sophonphan J, Wacharachaisurapol N, Jitrungruengnij N, Puthanakit T. Risk factors of severe hospitalized respiratory syncytial virus infection in tertiary care center in Thailand. *Influenza Other Respir Viruses*. 2021; 15(1): 64–71. https: doi:10.1111/irv.12793
3. Chen L, Miao C, Chen Y, Han X, Lin Z, Ye H, et al. Age-specific risk factors of severe pneumonia among pediatric patients hospitalized with community-acquired pneumonia. *Ital J Pediatr*. 2021; 47(1): 100. https://doi.org/10.1186/s13052-021-01042-3

1. Lingshuang Ren, Li Lin, Hua Zhang and Qianli Wang should be considered joint first author. Hongkai Lian, Jiangtao Wang and Hongjie Yu should be considered joint senior author. [↑](#footnote-ref-1)
